# Thymoquinone recovers learning function in a rat model of Alzheimer’s disease 

**Published:** 2018

**Authors:** Parvin Poorgholam, Parichehreh Yaghmaei, Zahra Hajebrahimi

**Affiliations:** 1 *Department of Biology, Science and Research Branch, Islamic Azad University, Tehran, Iran*; 2 *Aerospace Research Institute, Ministry of Science Research and Technology, Tehran, Iran*

**Keywords:** Alzheimer’s disease, Thymoquinone, Rat, Amyloid beta, Nigella sativa

## Abstract

**Objective:**

Alzheimer's disease is a neurodegenerative disorder characterized by accumulation of amyloid beta in the hippocampus. In recent decades, herbal medicine has been widely used to treat many neurodegenerative disorders,as in comparison to conventional drugs, herbal remedies exert minimal side effects. Here, the effects of thymoquinone, as the main active component of *Nigella sativa*, on passive avoidance memory in rat model of Alzheimer’s disease, were evaluated.

**Materials and Methods:**

Hippocampal injection of amyloid beta (Aβ) was used to induce Alzheimer’s disease in male Wistar rats, followed by intra peritoneal administrations of 5 and 10 mg/kg thymoquinone on a daily basis for 4 weeks. Animals were subjected to fear learning behavior in passive avoidance test and histopathological analysis of the hippocampus was done. Shuttle box test was used to evaluate the condition studying memory. Thioflavin-S and Hematoxylin and Eosine staining were done to confirm Aβ plaque formation and to evaluate the effect of thymoquinone on the pyramidal cells in the hippocampal CA1 region.

**Results:**

Amyloid beta caused cognitive dysfunction reflected by increasing initial and step-through latency along with plaque formation and degeneration of pyramidal cells in the hippocampus. Thymoquinone administration ameliorated this effect by significant reductions in plaque formation in CA1 region of the hippocampus and increased latency time. It also increased the number of surviving neurons in the hippocampus.

**Conclusion:**

It seems that thymoquinone improved learning function in a rat model of Alzheimer’s disease. Thus, thymoquinone could be possibly used as an anti-neurodegenerative agent for protecting hippocampal neurons against neurotoxic effects of Aβ in patients with Alzheimer’s disease.

## Introduction

Alzheimer's disease (AD), also known as just Alzheimer’s was first introduced by Dr. Alois Alzheimer in 1907. It is the common form of dementia that accounts for 60 to 70% of dementia cases among elderly people (Burns and Iliffe, 2009 ▶). It is an irreversible and progressive neurodegenerative disorder characterized by memory loss, cognitive dysfunction, and deterioration of thinking skills and other behavioral abilities that interferes with daily tasks of life (Mount and Downton, 2006 ▶).

AD is associated with brain changes and formation of abnormal structures called plaques and tangles which attribute to neuron damage and death. Plaques are extracellular accumulations of a protein fragment called amyloid beta (Aβ). Amyloid-beta comes from a larger protein found in the fatty membrane surrounding nerve cells. It is produced from the amyloid precursor protein (APP) that is cleaved by beta- and gamma-secretase to form Aβ which is chemically sticky and gradually builds up into plaques. A mutation in APP or in the catalytic subunit of γ-secretase (presenilin-1 or presenilin-2) can lead to early onset of AD (Lee et al., 2001 ▶). It is likely that plaques are produced as a result of an imbalance between the production and elimination of Aβ which impair synapses in a way that signals cannot be transferred between nerve cells (Kehoe et al., 2009 ▶). Tangles are twisted fibers of hyper phosphorylated tau protein (p-tau) accumulated inside the nerve cells. They kill nerve cells by preventing the normal transport of food and energy around the brain cells (Saido, 2013 ▶; Belluti et al., 2013 ▶).

Oxidative stress is considered responsible for neurodegenerative disorders such as Alzheimer’s disease (Perry et al., 2002 ▶). Some studies have been shown that Aβ induces the production of hydrogen peroxide, lipid peroxide, superoxide and inflammatory cytokines in the brain (Esposito et al., 2006 ▶; Huang et al., 1999 ▶). Also, studies have shown that in the brain of patients with Alzheimer’s disease, there are lower concentrations of acetylcholine (ACh). This neurotransmitter helps to transfer messages between certain nerve cells and its function is important for processing memory and learning (Francis, 2005 ▶; Kihara and Shimohama, 2004 ▶; McGleenoon et al., 1999 ▶). It has been demonstrated free radicals reduce Ach levels in the hippocampus through degeneration of cholinergic neurons (Vinod et al., 2009 ▶). There are also claims that acetylcholinesterase (AChE), the enzyme which inactivates acetylcholine at the synaptic clefts, is increased around plaques (Sberna et al., 1997 ▶). Therefore, inhibition of AChE resulting in prevention of the normal breakdown of ACh, may compensate for depletion of Ach seen in Alzheimer's disease.

In recent decades, herbal medicine is widely used to treat many neurodegenerative disorders, as herbal remedies exert minimal side effects, marked availability and ease of administration (Selvam, 2008 ▶; Lobo et al., 2010 ▶). *Nigella sativa* (*N. sativa*), also known as black seed, black cumin or kalonji seeds is an annual plant belonging to Ranunculaceae family. It is native to Southern Europe, North Africa, South and Southwest of Asia, and is very popular among people of Mediterranean countries (Houghton et al., 1995 ▶). It has been used by people for thousands of years for the treatment of asthma, cough, bronchitis, headache, rheumatism, fever, and influenza (Ali and Blunden, 2003 ▶). Anti-inflammatory, anti-oxidative and neuroprotective effects of *N. sativa* have been suggested (Houghton et al., 1995 ▶; Burits and Bucar, 2000 ▶; Kanter et al., 2006 ▶). It has been shown that the extracts of *N. sativa* protect frontal cortex and brain stem from toluene-induced degeneration in rats (Kanter, 2008a ▶, 2008b, ▶ 2011). Studies have demonstrated that thymoquinone (TQ), the active compound of *N. sativa*, has acetylcholinesterase inhibitory effect and protects cultured rat primary neurons against Aβ-induced neurotoxicity (Kanter, 2011 ▶; Alhebshi et al., 2013 ▶). With respect to the above-mentioned evidence, the aim of the present work was to investigate the effects of TQ on Aβ formation in a rat model of Alzheimer’s disease. Also, TQ neuroprotective effects on learning function were evaluated.

## Materials and Methods

**Drugs**

Amyloid beta (Aβ_1-42_), TQ and thioflavin-S were obtained from Sigma Chemical Co. (St. Louis, MO, USA). In this study, 50 µg of the Aβ was dissolved in 50 µl sterile phosphate buffered saline (PBS) solution, followed by incubation at 37ºC for 1 week to allow fibril formation and then, kept at -21°C before use. Moreover, TQ was dissolved in tween to prepare doses of 5 and 10 mg/kg. Ketamine (10%) and xylazine (2%) were purchased from Alfasan Co. (Holland). 

**Animals and treatments**

Thirty adult male Wistar rats (8-10 weeks old; 200-250 g), were purchased from Pasteur Institute (Tehran, Iran). Animals were kept in the animal house of Islamic Azad University, science and Research Branch, under standard laboratory conditions of humidity and temperature with 12-hr light: 12-hr dark cycles. They had unlimited access to food and water. Animals were cared in accordance with the guidelines for the Care and Use of Laboratory Animals (Committee for the update of the guide for the care and use of laboratory animals, 1996). All protocols were approved by the Animal Care and Use Committee of Islamic Azad University, science and Research Branch, Tehran, Iran, and all experiments were carried out between 10 a.m. and 5 p.m. All efforts were made to minimize animal suffering and to reduce the number of animals used.

Animals were randomly divided into five groups (n=6) as follows: 1- control group that received distilled water without surgery (no treatment); 2- PBS treatment group which underwent sham operation and received PBS as Aβ solvent by stereotaxic surgery; 3- tween + Aβ treatment group which was injected with Aβ bilaterally and received intra peritoneal injection of tween (80%) as TQ solvent for 4 weeks after recovery from stereotaxic surgery; 4-experimental group 1 (EXP-1) which was injected with Aβ bilaterally and received intra peritoneal injection of TQ(5 mg/kg) for 4 weeks; and 5- experimental group 2 (EXP-2) which was injected with Aβ bilaterally and received intra peritoneal injection of TQ(10 mg/kg) for 4 weeks. 

For the stereotaxic procedure, animals were deeply anesthetized by intra-peritoneal injection of ketamine (80 mg/kg) and xylazine (10 mg/kg) (Alfasan, Woerden, and Holland) and positioned in the Stoelting stereotaxic device (incisor bar set at -3.3 mm) (USA). The scalp of the animals was shaved and cleaned with povidone-iodineand ethanol to minimize the risk of infection and incision was made on the midline. The cranium drilled through the skull and bilateral injection of 2 µl of Aβ(to groups 3, 4 and 5) or PBS (to group 2) was done into the right and left dorsal hippocampus (2µl in each side) according to a Paxinos and Watson atlas (Paxinos and Watson 1986 ▶): -3.5 mm posterior to bregma, 2 mm lateral to sagittal suture, and 2.8 mm below dura. Each injection was done within 60-90 sec by a 5-µl Hamilton microsyringe and then the syringe was kept in place for 5 min to ensure complete injection of drug (Wu et al. 2007 ▶). After complete diffusion of the drug, the syringe was withdrawn slowly. During surgery, body temperature was maintained at 37°C by controlling the room temperature by a heating system. After surgery, animals were kept in a temperature and humidity-controlled chamber and allowed to recover from anesthesia before returning to the cage. The rats were monitored routinely and given postoperative care for 7 days to prevent infection and reestablish of full spontaneous feeding. Behavioral tests were performed 5 weeks after the surgery, during 2 days.

**Passive avoidance test**

Shuttle box test was used to evaluate the condition studying memory of the rats. The device (60 cm long, 18 cm wide and 18 cm high) consisted of one illuminated chamber and one dark chamber which were connected through a guillotine door. Electrical shocks could be transferred by a separated stimulator to the grid floor of the apparatus. The test was performed for 2 consequently days. On the first day, animals were allowed to explore both chambers. For habituation, rats were placed in the illuminated area and allowed to explore for 5 sec. After 5 sec, the door was lifted and the rats were allowed to enter the dark compartment. When the animal entered the dark chamber, the door was closed, and the latency to enter was recorded. Animals with a latency period greater than 100 sec were excluded from the study. After 10 sec, the rats were returned to the home cage. Training was done 30 min after habituation. For training, the rats were put in the illuminated chamber again and allowed to explore for 5 sec. After that, the door was lifted. When the rats entered the dark area, the door was closed and a foot shock (1 mA for 5 sec) was induced. 20 sec after the electrical shock, animals were placed in the home cage. In order to test their learning, the rats were then placed back in the compartment after 2 min and their latency (initial latency; t1) to enter dark chamber was recorded. The training test (electrical shock) was repeated if the latency period was <120 sec. The maximum number of training considered for each animal was three times. On the following day, in order to test their short-term learning, their latency (as step-through latency) to cross through the gate between the compartments was measured while no shock was being given to the rats. The maximum procedure time was 300 sec in this study (Akhondzadeh and Abbasi 2006 ▶; Sarkaki et al. 2013 ▶; Everss and Parra 1998 ▶).

**Histological procedure**

After passive avoidance analysis, rats were killed and their brains were removed and evaluated for histological changes and Aβ plaque formation. Samples were fixed in 10% paraformaldehyde for 24 hr and then embedded in paraffin after standard processing of dehydration and clearing. Then, the brains were sectioned with 6-μm thickness (from Bregma 2–4 mm of frontal cortex, _2.5 mm to _4.5 mm of the hippocampal formation and _10 mm to _15 mm of the cerebellar cortex), mounted on glass slides and stained with Hematoxylin and Eosine (H&E) and thioflavin-S (Suvarna et al. 2008 ▶). For thioflavin-S staining, the sections were immersed in -1% thioflavin-S for 5 min at room temperature, washed with tap water, and dried at room temperature; strong green fluorescence of thioflavin-S was observed using aconfocal UV microscope. For quantitative analysis, the percentage of plaque area/number of plaques, was calculated by using the ImageJ analysis program.

**Statistical analysis**

SPSS statistical software (version 17) was used for data analyzing. All data are presented as means±S.E.M. One-Way ANOVA (one way analysis of variance) with Tukey test was used for analyzing the data from shuttle box test and comparison of different group means. A p<0.05 was considered statistically significant. 

## Results

**Learning abilities**

**Figure 1:**
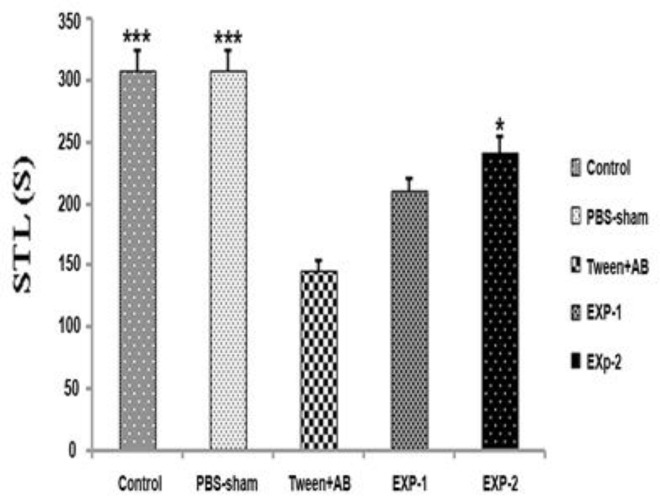
Initial latency period obtained in the passive avoidance test. Values are expressed as means±SEM. Control, PBS-sham (bilateral hippocampal injection of PBS), tween+Aβ (bilateral hippocampal injection of Aβ which received i.p. injection of tween), EXP-1 (bilateral hippocampal injection of Aβ which received 5mg/kg of TQ i.p.), and EXP-2 (bilateral hippocampal injection of Aβ which received 10mg/kg of TQ i.p.). *p<0.05 and ***p<0.001 as compared to tween+Aβ group

Passive avoidance test was used to evaluate the effects of TQ on learning function in an Aβ-induced rat model of Alzheimer’s disease. Figure 1 illustrates the initial latency period in rats on the first day of passive avoidance test (learning) concerning the move from the illuminated area of shuttle box to dark zone, 2 min after the first electrical shock. As indicated in Figure 1, the initial latency time of tween+Aβ treated group was significantly decreased compared tothe control and PBS-sham operated groups (p<0.001). In this experiment, treatment with TQ for 28 days improved learning performance. Initial latency time in rats with Aβ-induced Alzheimer's that received TQ(10 mg/kg; ip) for 4 weeks (EXP-2 group) was obviously longer than the tween+Aβ operated group (p<0.05). There was no significant difference among the tween+Aβ operated group and experimental group number 1 (EXP-1; Aβ induced Alzheimer rats which received intra peritoneal injection of thymoquinone (5 mg/kg) for 4 weeks). Regarding initial latency data, thymoquinone treatment at the dose of 10 mg/kg for 4 weeks may improve the learning function of Alzheimer’s rat (p<0.05). 

**Figure 2:**
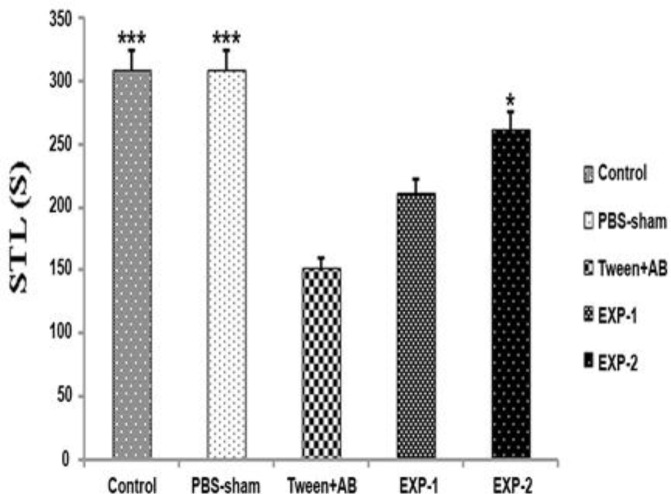
Step-through latency period obtained in a passive avoidance test. Values are presented as means±SEM. Control, PBS-sham (bilateral hippocampal injection of PBS), tween+Aβ (bilateral hippocampal injection of Aβ which received i.p. injection of tween), EXP-1 (bilateral hippocampal injection of Aβ which received TQ 5mg/kg i.p.), and EXP-2 (bilateral hippocampal injection of Aβ which received TQ 10mg/kg i.p.). *p<0.05 and ***p<0.001 as compared to tween + Aβ treated group

Figure 2 shows the step-through latency in rats on the second day of passive avoidance test regarding the move from the illuminated area of shuttle box to dark zone where no shock was given to the rats. Similarly, the step-through latency time in tween+Aβ operated group was significantly decreased compared to the control and PBS-sham operated groups (p<0.001). Thus, treatment with TQ for 28 days improved learning function in the rats with Alzheimer’s disease. Step-through latency period in EXP-2 group was obviously greater than that of the tween+Aβ operated group (p<0.05). There was no significant difference between the tween+Aβ operated group and EXP-1 group. Regarding step-through latency period, TQ treatment at the dose of 10 mg/kg for 4 weeks could improve the short-term passive avoidance learning performance of rats with Alzheimer’s disease(p<0.05).

**Histological results**

**Thioflavin-S staining**

**Figure 3:**
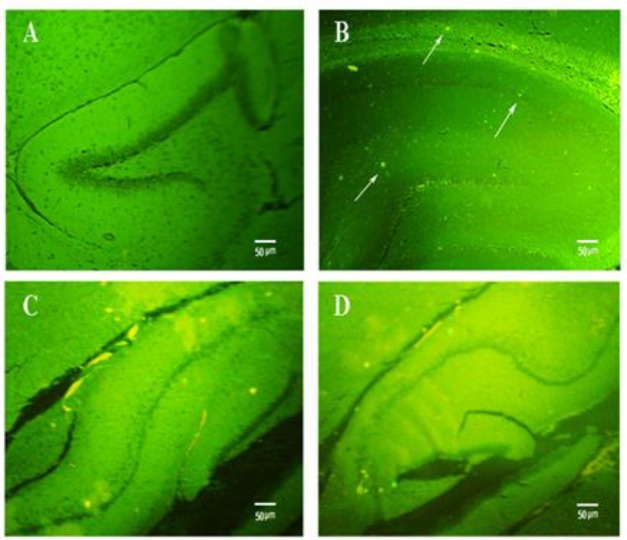
Detection of Aβ plaques in the hippocampus of A: control, B: tween+Aβ, C: EXP-1 and D: EXP-2 groups using thioflavin-S staining (X40). White arrows show Aβ plaques in tween+Aβ group. Administration of TQ obviously decreased the number of plaques in EXP-1 and EXP-2 groups. Evidently, TQ at the dose of 10 mg/kg (EXP-2 group) further decreased the number of Aβ plaques compared to the EXP-1 group (treated with TQ 5 mg/kg). The number of plaques intween + Aβ, EXP-1, and EXP-2 groups were 34±3.00, 19.6±2.23 and 12.2±1.32, respectively

Bilateral hippocampal injection of Aβ was used to develop Alzheimer’s disease in rats. Thioflavin-S staining was done to confirm Aβ plaque formation after 37-day Aβ injection (postoperative care: 7 days, TQ treatment: 4 weeks, Passive avoidance test: 2 days). Comparison of brain slides from different groups showed obvious histological changes in the hippocampus. No plaque formation was detected in the control rats (Figure 3A) and PBS-sham operated group (data not shown). The control and PBS-sham hippocampus showed to be clean of the green plaques, while other groups showed the presence of Aβ plaques. These results indicated that Aβ treatment can be used toinduce Alzheimer’s disease in rats. 

Furthermore, 28-day treatment of EXP-1group with TQ 5 mg/kg, dramatically reduced the number of Aβ plaques in hippocampus sections (Figure 3C; p<0.05). Increasing the concentration of TQ to 10 mg/kg caused further reduction in plaques in the hippocampus of EXP-2 group (Figure 3D; p<0.05). The number of plaques in tween+Aβ, EXP-1, and EXP-2 groups were 34±3.00, 19.6±2.23 and 12.2±1.32, respectively (Table 1). This observation may indicate that TQ clears Aβ plaques in a dose-dependent manner. 

**Hematoxylin and Eosine (H&E) staining**

**Figure 4:**
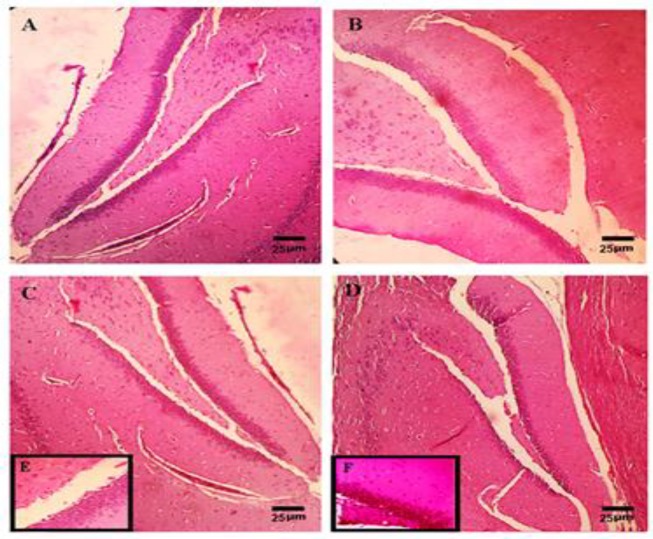
Hematoxylin and eosin staining revealed the hippocampus of the control rats (X100) and hippocampal neuronal loss of the tween + Aβ rats after 35 days of Aβ injection (B, X100). Treatment of animals with Alzheimer's with TQ improved the number of surviving pyramidal cells in the hippocampal CA1 region in the EXP-1 (C: X100 and E: X400) and EXP-2 (D: X100 and F: X400) groups. The number of surviving neurons in EXP-2 group (treated with 10 mg/kg) was markedly higher than EXP-1 group (treated with TQ 5 mg/kg). The mean of total number of CA1 neurons in control, tween + Aβ, EXP-1 and EXP-2 groups were 451.75±38.54, 67.50±6.19, 141.25±10.05 and 311.75±34.94; respectively

H&E staining was performed to evaluate the effect of TQ on the pyramidal cells in the hippocampal CA1 region. The round cells with big, with intact cytoplasmic membrane and regular nuclei without any nuclear condensation or distorted aspect, were considered live. The pyramidal cells in the hippocampal CA1 region, in the control (Figure 4A) or PBS-sham operated group (data not shown), were seen at high magnifications and lined up regularly. The mean of total number of CA1 neurons in control group was 451.75±38.54 (Table1). As shown in Figure 4B, bilateral hippocampal injection of Aβ considerably decreased the number of neurons in the tween+Aβ operated group (67.50±6.19; p≤0.001).

 In this study, TQ increased the number of surviving pyramidal cells in the hippocampal CA1 region of EXP-1 group (Figures 4C and 4E) which received TQ 5 mg/kg for 28 days (141.25±10.05; p≤0.01). Increasing the concentration of thymoquinone to 10 mg/kg further enhanced the survival rate of pyramidal cells (311.75±34.94; p≤0.001) in the hippocampus of EXP-2 group (Figures 4D and 4F).

**Table1:** The mean of total number of Aβ plaque and neurons in the hippocampal region of different groups

**Number**	**Control**	**Tween-sham**	**EXP-1**	**EXP-2**
**Plaque**	-	34±3.00	19.6±2.23*	12.2±1.32**
**Neuron**	451.75±38.54	67.50±6.19	141.25±10.05*	311.75±34.94**

Values represent mean±S.E.M.

* p<0.01 (vs. tween+Aβ group),

** p<0.001 (vs. tween+Aβ group).

## Discussion

Alzheimer's disease is a neurodegenerative disorder characterized by accumulation of Aβ in the hippocampus leading to impaired memory and cognitive function in elderly people (Burns and Iliffe, 2009 ▶; Mount and Downton, 2006 ▶; Kehoe et al., 2009 ▶). The objective of the present study was to examine neuroprotective effects of TQ in a model of Alzheimer’s disease in male Wistar rats. Aβ was used for induction of Alzheimer's disease in rats (Azad et al., 2011 ▶; Baluchnejadmojarad et al., 2011 ▶; Esposito et al., 2006 ▶; Huang et al., 1999 ▶; Takata et al., 2007 ▶). 

In the present work, injection of Aβ impaired hippocampal learning function in tween+Aβ treated group as compared to control and PBS-sham groups. Passive avoidance test showed a significant reduction in initial latency and subsequently in step-through latency of tween+Aβ treated rats that indicates learning deficit and reflects hippocampal neurodegeneration. We also histologically studied the brain in different groups. The results confirmed the production of Aβ plaques in CA1 region of the hippocampus of tween+Aβ treated rats, 37 days after Aβ treatment. Strong green fluorescent plaques were observed in the hippocampal sections of Aβ-treated rats following staining with thioflavin-S. Furthermore, H&E staining implicated a considerable reduction in neuron numbers in the same region which also demonstrated neural degeneration. These results confirm that Aβ injection caused amnesia in rats; therefore, it can potentially be used to develop a laboratory model to study cognition. 

Thymoquinone in this study restored learning function of the hippocampus. Our data revealed that TQ was able to improve the cognitive dysfunction induced by Aβ. Both initial latency and step-through latency periods were increased in rats with Alzheimer's disease treated with TQ. TQ is one of the important phenolic components of *N. sativa*. Some therapeutic properties of *N. sativa *including anti-inflammatory, anti-oxidative and neuroprotective effects have been shown (Houghton et al., 1995 ▶; Burits and Bucar, 2000 ▶; Kanter et al., 2006 ▶). Anti-neurodegenerative effects of *N. sativa* and TQ have been shown by various studies. Kanter examined the effects of 12-week administration of *N. sativa* and TQ on neural injury in the frontal cortex, brain stem and hippocampus of rats treated with toluene and found no histopathological alterations in the brain of treated groups (Kanter 2008a ▶, 2008b ▶, 2011). It is clear that accumulation of Aβ is involved in many features of Alzheimer’s disease. *In vitro* study of Alhebshi et al. (2013) ▶ showed that TQ could increase the number of surviving cells in cultured hippocampal neurons after exposure to a neurotoxic concentration of Aβ. 

In the present study, TQ 5 and 10 mg/kg were used. The dose of 5 mg/kg did not cause any significant improvement in learning and cognitive function of rats with Alzheimer's disease. In contrary, a higher dose of TQ (10 mg/kg) significantly restored initial latency and step-through latency periods EXP-2 group, suggesting that TQ improves learning function in a dose-dependent manner. These observations were also confirmed by histological analysis. The number of Aβ plaques in the hippocampus of rats with Alzheimer's disease considerably reduced after administration of TQ 10 mg/kg. Also, H&E staining of CA1 region of the hippocampus revealed that TQ improves pyramidal cell viability and protects hippocampal neurons against Aβ toxicity in EXP-2 group that are in line with the results of Alhebshi et al. (2013) ▶. Imam et al. (2016) ▶ examined the protective effects of black seed oil (extraction of *N. sativa *seeds) on cognitive function and cortico-hippocampal neural alterations in male Wistar rats. In that study, cognitive impairment was induced by scopolamine in rats and Morris water maze and Y maze tests were used for evaluation of cognitive effect of black seed oil. Scopolamine is a chemical that can impair learning and memory in humans and rats. Black seed oil improved memory dysfunction and reversed cognitive impairment induced by scopolamine by reducing latency period in the Morris water maze test. These results emphasize that *N. sativa* is able to restore memory and cognitive performance of the hippocampus. Bin Sayeed et al. (2013 ▶ and 2014) also reported therapeutic potential of black seed oil on memory, attention, and cognition, in rats with scopolamine-induced amnesia. 

The protective effects of TQ against Alzheimer’s disease are most likely due to its AChE inhibitory and antioxidant properties. Reduction in acetylcholine levels as well as increased levels of acetylcholinesterase activity in the hippocampus of patients with Alzheimer’s, has been demonstrated (Francis, 2005 ▶; Kihara and Shimohama, 2004 ▶; McMgleenon et al., 1999 ▶; Vinod et al., 2009 ▶; Sberna et al., 1997 ▶). Therefore, inhibition of AChE is a therapeutic approach in Alzheimer’s disease. Jukic et al. (2007 ▶) studied the properties of TQ isolated from an aromatic plant, *Thymus vulgaris* L. and found that TQ can inhibit AChE activity *in vitro*. 

Furthermore, oxidative stress is the major inducer of neurodegenerative disorders including Alzheimer’s disease (Perry et al., 2002 ▶; Esposito et al., 2006 ▶; Huang et al., 1999 ▶). Al-Majed et al. (2006) ▶ induced oxidative damage in the hippocampus of rats through ischemia by bilateral occlusion of carotid arteries and administrated TQ for 12 days (5 days before ischemia and during 7 days of reperfusion). They reported that treatment with TQ could increase glutathione levels, catalase, and superoxide dismutase activities while it decreased malondialdehyde contents, indicating anti-oxidative functions of TQ. 

In Conclusion, our data revealed that hippocampal injection of Aβ could induce Alzheimer’s disease in male Wistar rats while administration of TQ could prevent learning dysfunction and improve initial latency and step-through latency. TQ also decreased plaque formation in CA1 region of the hippocampus while increased the number of surviving neurons and protected pyramidal cells against neurotoxic effects of Aβ. Therefore, TQ and *N. sativa* can be used as protective agents and/or adjunct treatments for age-related neurodegenerative disorders such as Alzheimer’s and Parkinson's disease. 
